# Supplementary material for: Oncofetal gene SALL4 reactivation by hepatitis B virus counteracts miR-200c in PD-L1-induced T cell exhaustion
Source: Nat Commun. 2018 Mar 28;9:1241. doi: 10.1038/s41467-018-03584-3 (PMC5871883; doi:10.1038/s41467-018-03584-3)
Supplement: Supplementary file 1 — Supplementary Information [file 41467_2018_3584_MOESM1_ESM.pdf]

Supplementary Information

Oncofetal gene *SALL4* reactivation by hepatitis B virus counteracts miR-200c in PD-L1-induced T cell exhaustion

Sun et al.

Supplementary Figures 2

Supplementary Figure 1 2
Supplementary Figure 2 4
Supplementary Figure 3 5
Supplementary Figure 4 6
Supplementary Figure 5 7
Supplementary Figure 6 8
Supplementary Figure 7 9
Supplementary Figure 8 10

Supplementary Tables 11

Supplementary Table 1 11
Supplementary Table 2 12
Supplementary Table 3 13
Supplementary Table 4 14

Supplementary Figures

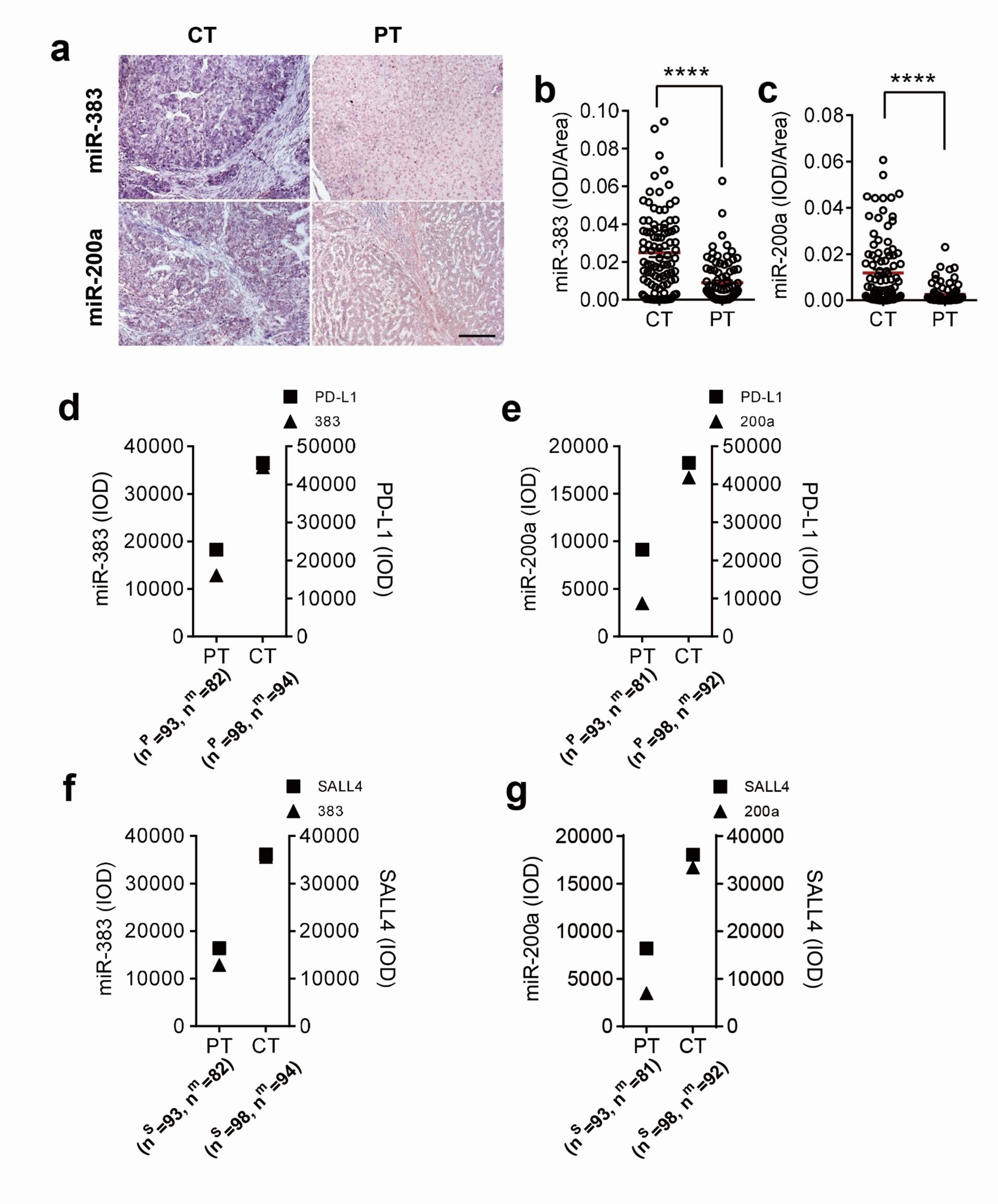

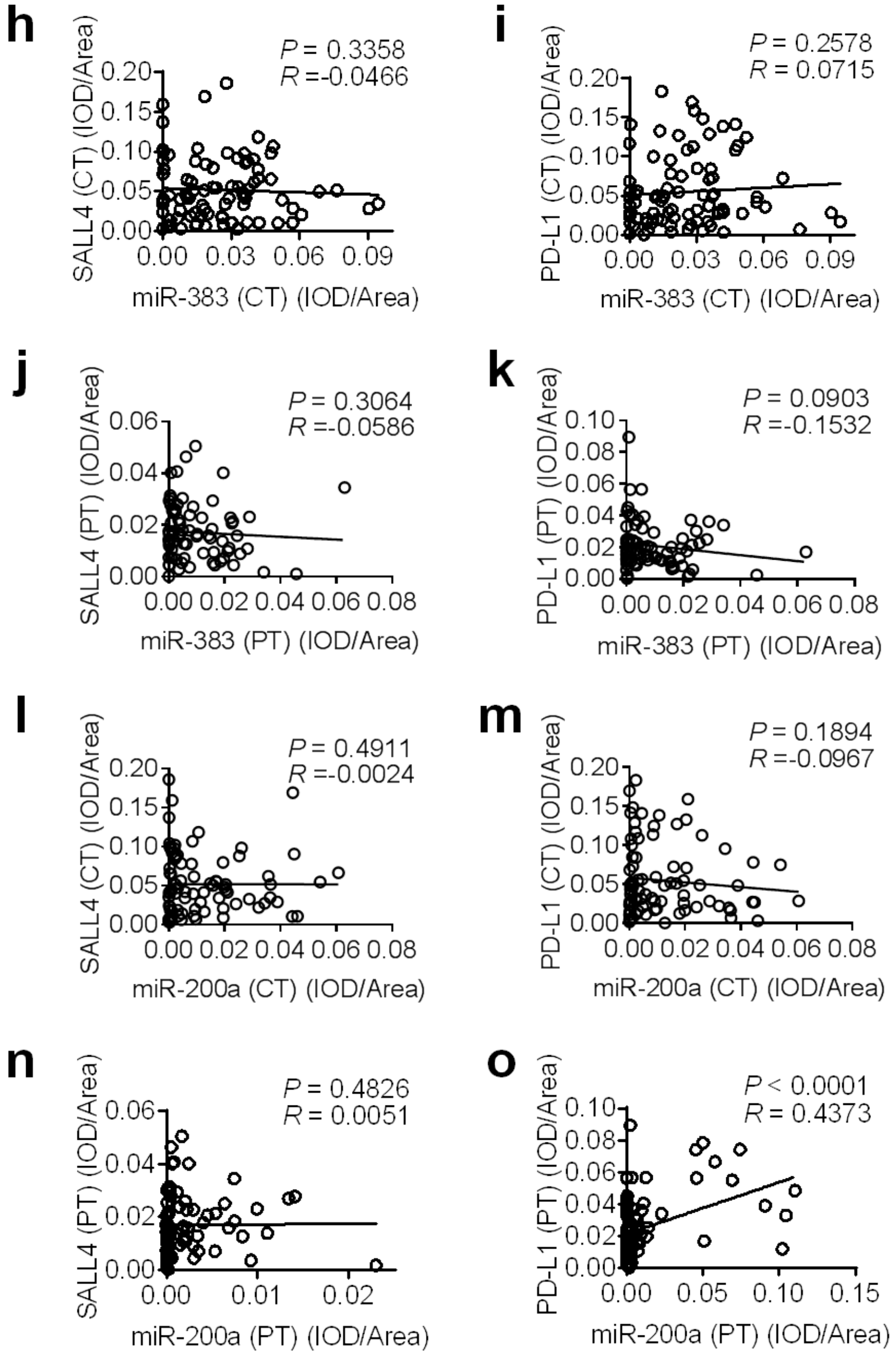

Supplementary Figure 1. Expression of other miR-200 family memebers in HCC patients. (a) *in situ* hybridization for hsa-miR-200a and hsa-miR-383 (blue purple colour) in CT and PT regions of HCC. Originalmagnifications: ×10, Bar = 200 µm. (b, c) Statistics summary of hsa-miR-383 (b, $P < 0.0001$) and hsa-miR-200a (c, $P < 0.0001$) expression in CT and PT regions. The results are expressed as the mean ± SEM. Cumulative data calculated by two-tailed unpaired Student's *t*-test. (d, e) Disparity analysis between miR-383 and PD-L1 (d) ormiR-200a and PD-L1 (e) in CT and PT regions. (f, g) Disparity analysis between miR-383 and SALL4 (f) ormiR-200a and SALL4 (g) in CT and PT regions. n^P, number of patients with PD-L1; n^m, number of patients withmiRNA; n^S, number of patients with SALL4. (h-k) Correlation analysis between miR-383 and SALL4 or PD-L1 inCT (h, i) and PT (j, k) regions of HCC. (l-o) Correlation analysis between miR-200a and SALL4 or PD-L1 in CT (l, m) and PT (n, o) regions of HCC. (h-o) Pearson's correlation coefficients and *P* values are shown.

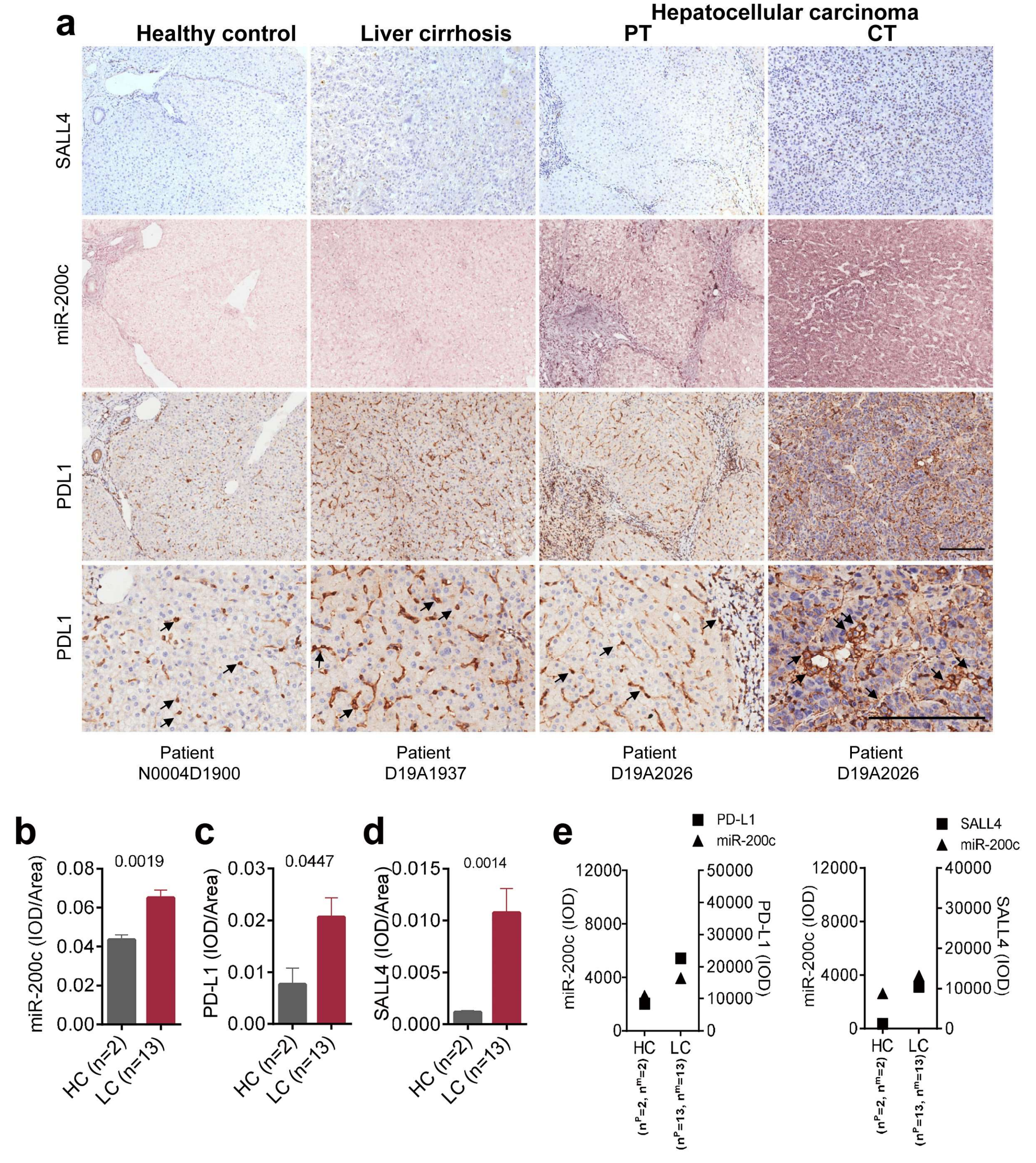

Supplementary Figure 2. miR-200c expression in patients with liver cirrhosis. (a) Representative images of SALL4 or PD-L1 immunostaining and miR-200c *in situ* hybridization from healthy control of liver (HC), liver cirrhosis (LC), and HCC. Original magnifications: ×10, ×40, Bar=200μm, 100μm. Statistical summary of theexpression of miR-200c (b), PD-L1 (c) and SALL4 (d). Cumulative data calculated by two-tailed unpairedStudent's *t*-test. (e) Disparity analysis between miR-200c and PD-L1 (Left) or miR-200c and SALL4 (Right) inHC (n=2) and LC (n=13).

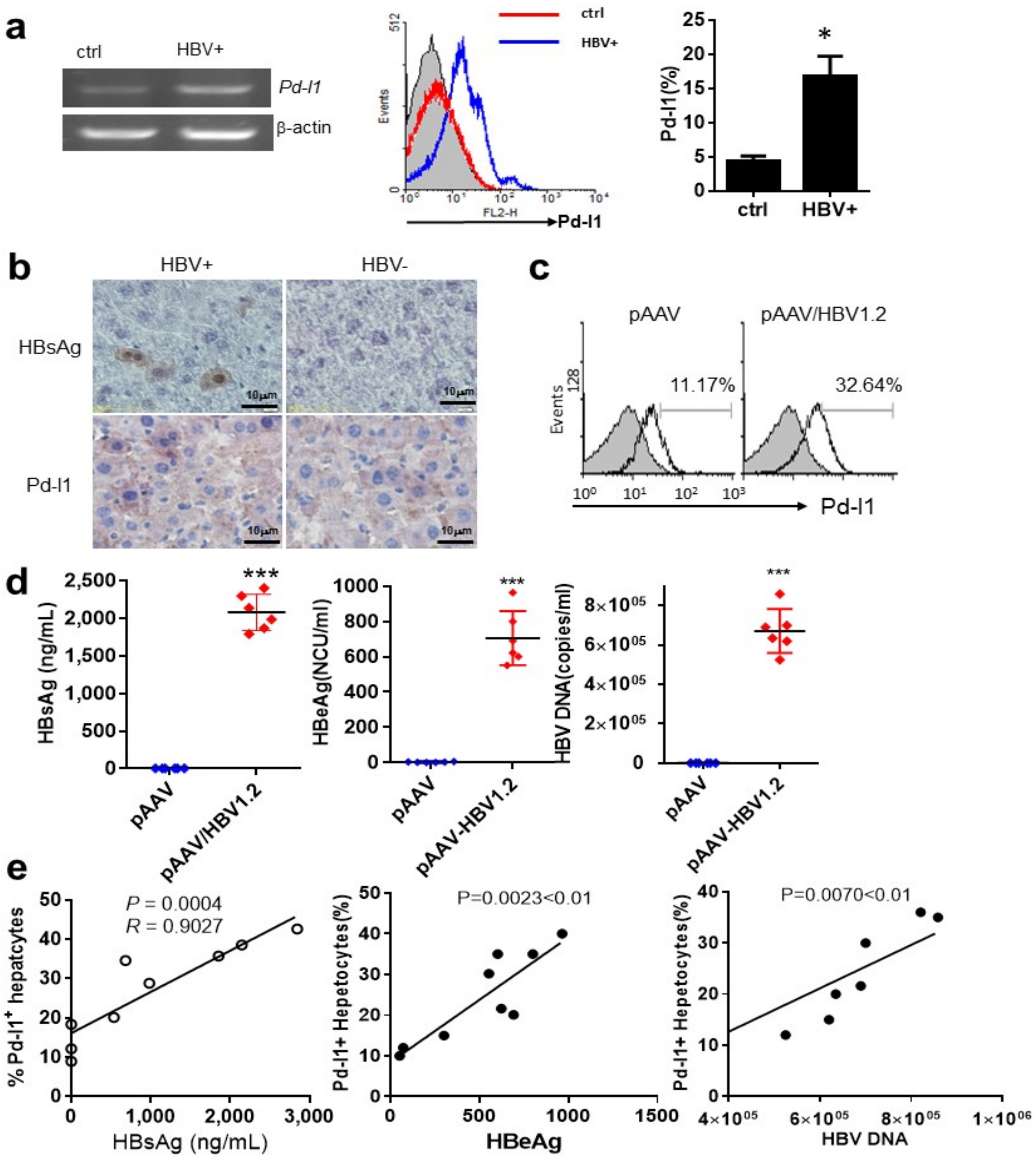

Supplementary Figure 3. HBV-induced PD-L1 expression on human and murine hepatocytes. (a) Comparison of mRNA and protein levels of PD-L1 on HLCZ01 (ctrl) and HBV^{+}HLCZ01 cells by PCR and FACS. (b-d) The plasmid pAAV/HBV1.2 (8 μg) was hydrodynamically injected into 4-5 week old C57BL/6 mice *via* the tail vein to obtain an HBV-persistent mouse model. Four weeks later, the HBsAg level in the serum was measured, and mice with 500 ng/mL of serum HBsAg level were defined as HBV-persistent mice. In HBV-persistent mice, the HBsAg and PD-L1 in liver tissue in HBV-persistent mice and HBV-negative (e.g. HBV-eliminated) mice was analyzed by immunohistochemistry (b). The PD-L1 on hepatocytes was analyzed by FACS (c). The serum levels of HBsAg and HBeAg were analyzed by ELISA, HBV-DNA level was analyzed by q-RT-PCR (d). The relationship between the PD-L1 on hepatocytes and the serum HBsAg, HBeAg or HBV DNA was analyzed (e). Data are expressed as the mean±SD from at least three separate experimentswith six mice per group. Significant differences calculated using unpaired two-tailed student's *t* tests. *$P < 0.05$, ***$P < 0.001$.

Gating strategy for Fig. 4a, b, e, f, Fig. 6g, h and Fig. 7c, e, g, h.

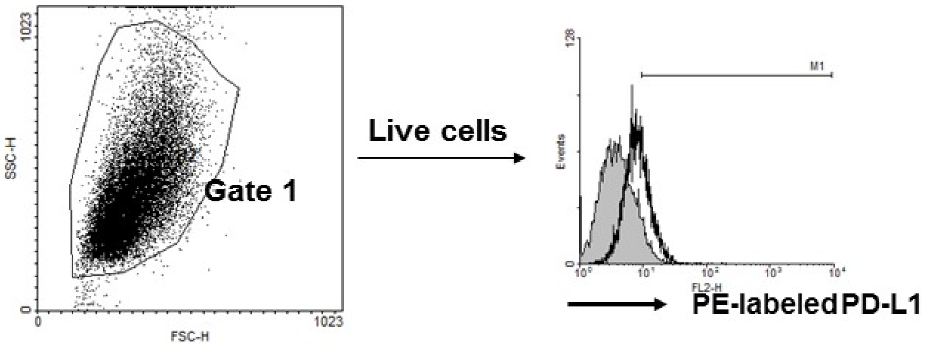

Gating strategy for Fig.5e

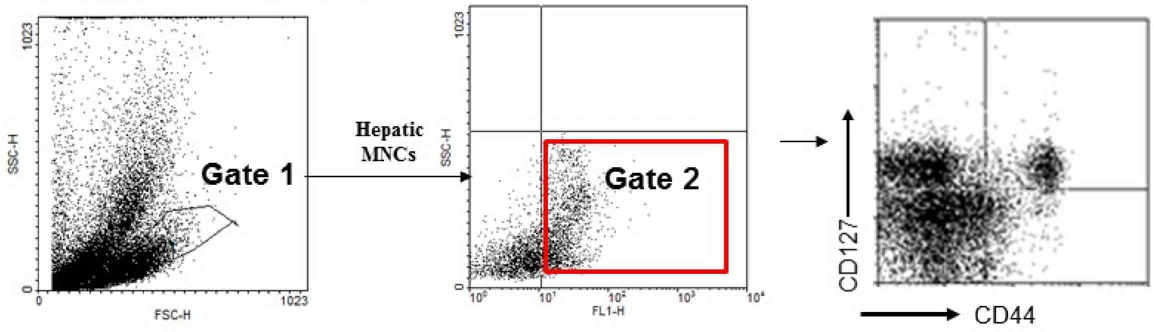

Gating strategy for Fig.5 f

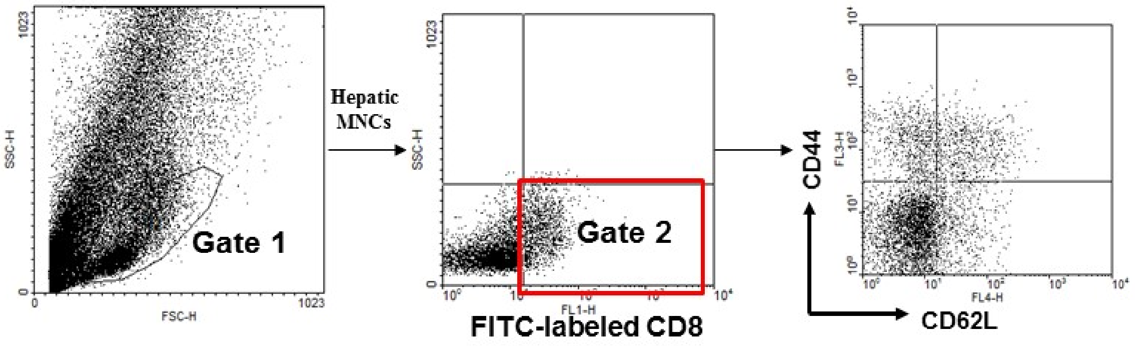

Supplementary Figure 4. Gating strategy for Fig. 4, Fig. 5, Fig. 6 and Fig. 7.

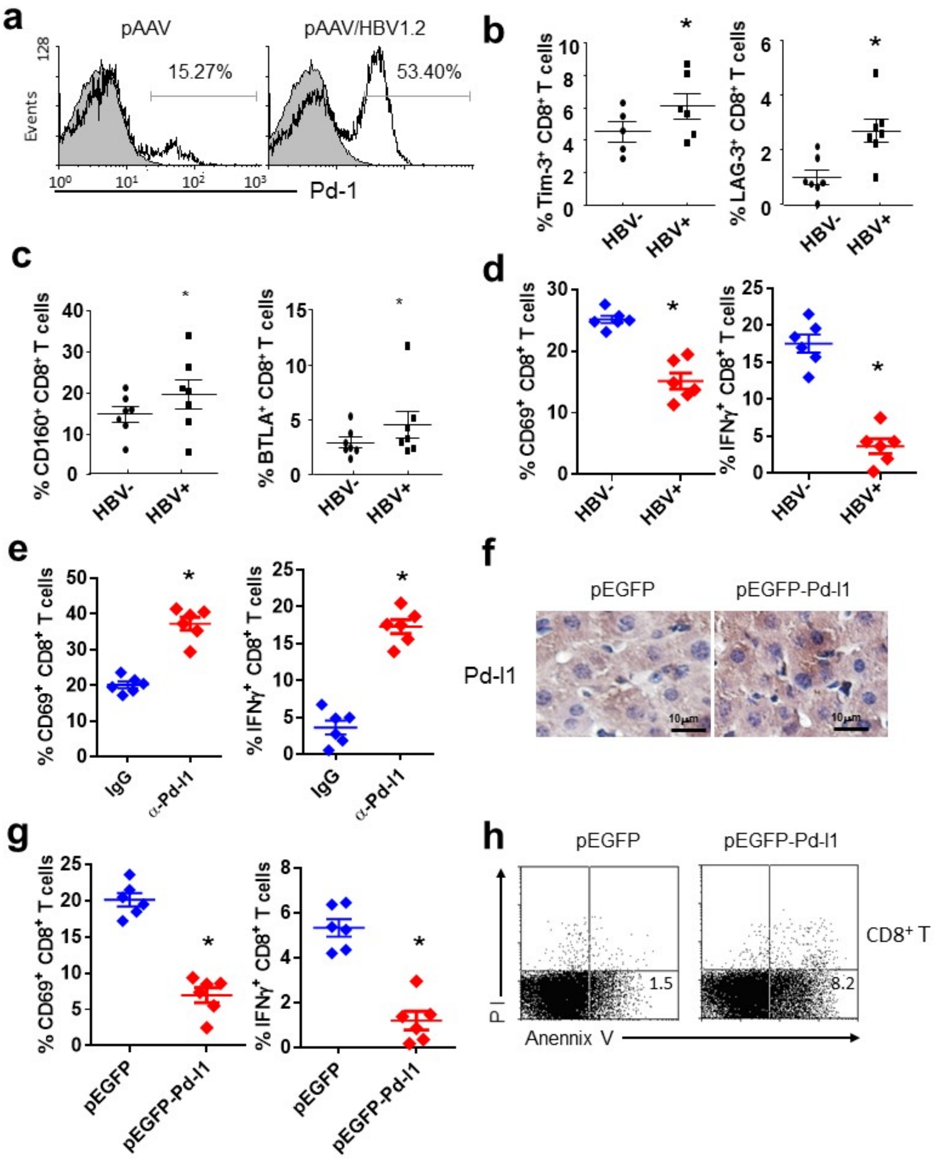

Supplementary Figure 5. HBV induces T cell exhaustion by up-regulating PD-L1 in HBV-persistent mice. (a-d) The plasmids pAAV/HBV1.2 (8 μg) and pAAV were hydrodynamically injected into 4-5 week old C57BL/6 mice *via* the tail vein, respectively; four weeks later, the percentage of hepatic Pd-1^{+}CD8^{+} T cells was analyzed by FACS (a), and the percentage of Tim-3 and LAG-3 (b), CD160 and BTLA (c), CD69^{+} and IFN-γ^{+} CD8^{+} T cells (d) was examined by FACS. (e) An anti-Pd-l1 Ab blockade was administered *i.p.* into HBV-persistent mice with IgG as a control; the percentage of liver CD69^{+} and IFN-γ^{+} CD8^{+} T cells was detected via FACS. (f, g) The pEGFP-*Pd-l1* expression vector was hydrodynamically injected into 5-6 week old C57BL/6 mice *via* the tail vein; four weeks later, the Pd-l1 level in liver tissue was analyzed *via* immunohistochemistry (f), the percentages of hepatic CD69^{+} and IFN-γ^{+} CD8^{+} T cells were detected *via* FACS (g), and the apoptosis of hepatic CD8^{+} T cells was analyzed *via* the double staining of Annexin V and PI and analyzed by FACS (h). The data are representative of three independent experiments with six mice per group. Significant differences calculated using unpaired two-tailed student's *t* tests. *P< 0.05, versus HBV- mice, IgG, or pEGFP.

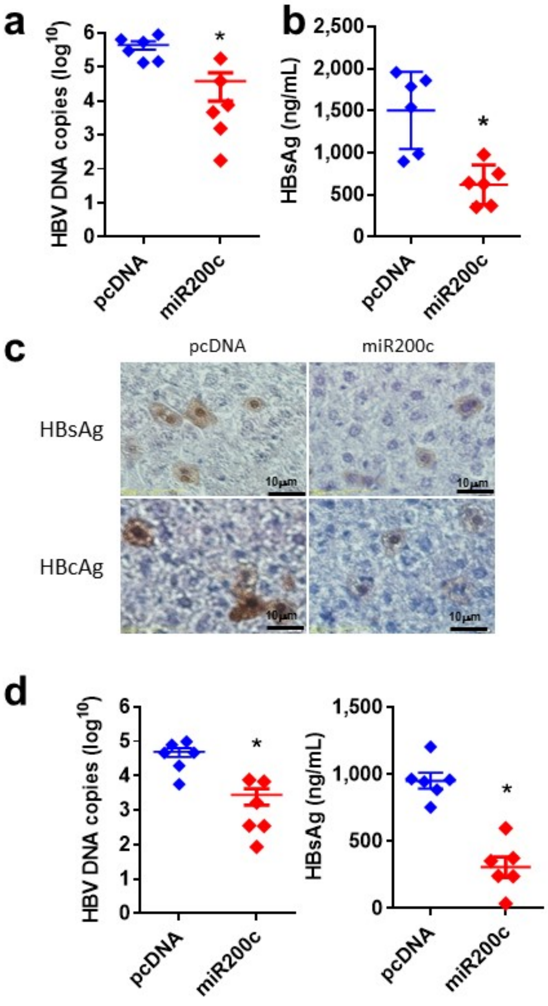

Supplementary Figure 6. miR-200c over-expression improves HBV elimination in HBV-persistent mice. (a-c) Serum HBV DNA in HBV-persistent mice after treated with miR-200c over-expression. HBV-persistent mice were hydrodynamically injected into 4-5 week old C57BL/6 mice via the tail vein; four weeks later, the serum HBV DNA was analyzed via q-RT-PCR (a), serum HBsAg was analyzed via ELISA (b), and the protein level of HBsAg and HBcAg in the liver tissue was analyzed via IHC (c). (d) pcDNA-miR-200c was hydrodynamically injected to mice one week prior pAAV/HBV1.2 hydrodynamic injection (HBV-persistence), the serum HBV DNA was analyzed by q-RT-PCR, and the serum HBsAg was analyzed via ELISA. Data are representative of three independent experiments with six mice per group. Significant differences calculated using unpaired two-tailed student's *t* tests. *$P < 0.05$, versus pcDNA.

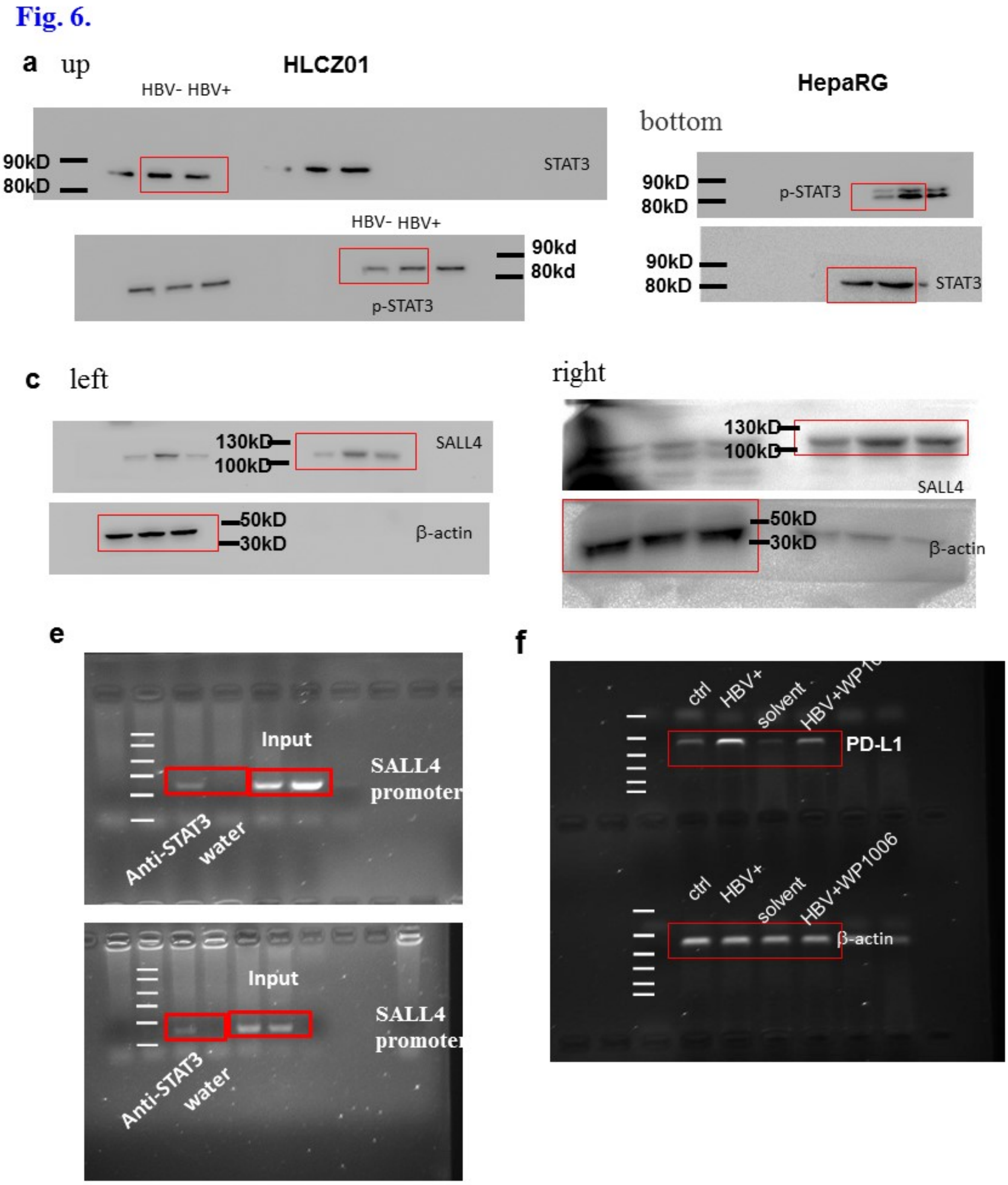

Supplementary Figure 7. Uncropped scans of blots shown in Fig. 6a, c, e, f.

Fig.7

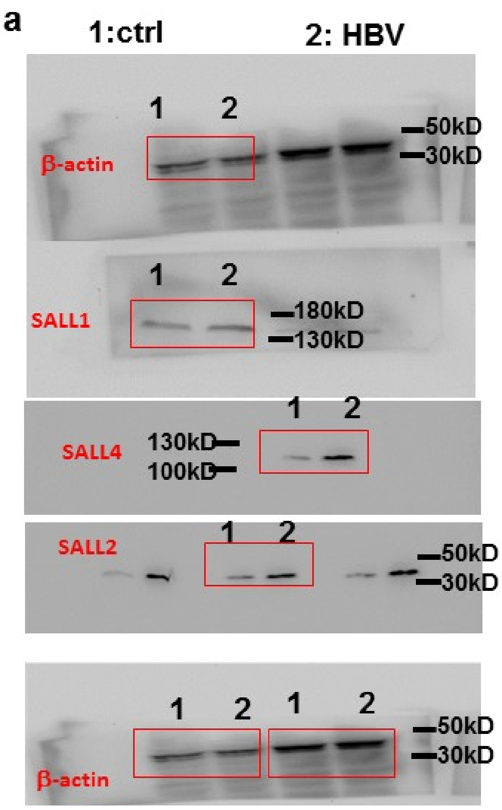

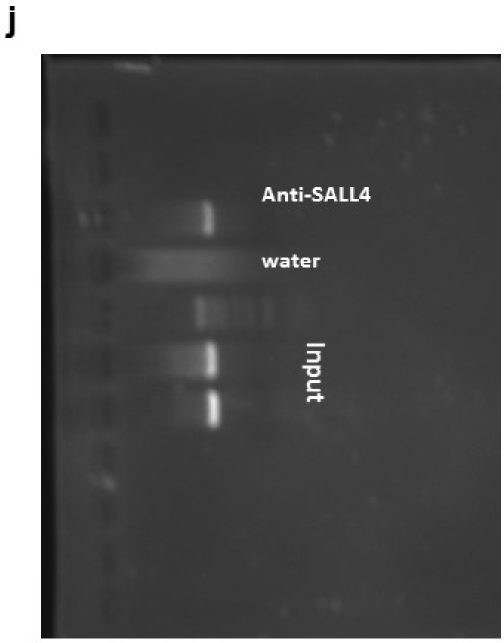

Supplementary Figure 8. Uncropped scans of blots shown in Fig. 7a, j.

Supplementary Table 1. Clinical Characteristics of the Enrolled Subjects.

Group	HCC	LC	HC
**Cases**	105	13	2
**Sex (male)**	92 (87.6%)	9 (69.2%)	2 (100%)
**Age (years)**	53.3 ± 10.2	48.5 ± 10.3	>20
**OS (months)**	33.2 ± 24	----	----
**Tumor Volume (cm3)**	394.3 ± 639.2	----	----
**Metastasis negative**	98 (93.3%)	----	----
**TNM-Ⅰ**	15 (14.3%)	----	----
**TNM-Ⅱ**	35 (33.3%)	----	----
**TNM-Ⅲ**	45 (42.9%)	----	----
**TNM-Ⅳ**	7 (6.7%)	----	----
**Cirrhosis positive**	105 (100%)	13 (100%)	----
**Survival**	33 (31.4%)	----	----

Overall survival (OS); Tumor-Node-Metastasis (TNM); Healthy control (HC); Liver cirrhosis (LC); Hepatocellular carcinoma (HCC).

Supplementary Table 2. Univariate Analysis of Survival Time in Patients with Liver Cancer.

	Overall Survival Time		
**Parameter**	**HR**	**95% CI**	***P*** *
**Clinical parameters**			
Gender (Male)	0.830	0.330 to 2.089	0.6927
Age (y)	0.988	0.962 to 1.014	0.3545
Metastasis (Y/N)	3.361	1.553 to 7.27	0.0021†
UICC (TNM) stage	2.050	1.363 to 3.083	0.0006†
Pathology Grading	1.287	0.820 to 2.020	0.2730
Tumor Volume	1.001	1.000 to 1.001	0.0167†
**Immune parameters**			
PD-L1^{CT}	1.010	1.005 to 1.015	<0.0001†
PD-L1^{PT}	1.008	0.991 to 1.026	0.3465
miRNA200c^{CT}	0.982	0.971 to 0.993	0.0020†
miRNA200c^{PT}	0.977	0.960 to 0.993	0.0058
SALL4^{CT}	1.013	1.007 to 1.019	<0.0001†
SALL4^{PT}	1.018	0.994 to 1.043	0.1405

NOTE: All categorical covariates were transformed into numeric codes before they were entered into the Cox model. Numeric codes are as follows: Gender: female = 1, male = 0. Metastasis: N = 0, Y = 1. Abbreviations: DFS, disease-free survival; OS, overall survival; HR, hazard ration; CI, confidence interval; UICC-TNM, International Union Against Cancer–TNM (staging systems);
†Significant.

Supplementary Table 3. Multivariate Cox Proportional Hazard Analysis for Survival Time among Patients with Liver Cancer.

	Overall Survival Time		
**Variable**	**HR**	**95% CI**	***P*** *
**Before backward selection**			
Metastasis (Y/N)	3.361	1.553 to 7.27	0.0021†
UICC (TNM) stage	2.050	1.363 to 3.083	0.0006†
Tumor Volume	1.001	1.000 to 1.001	0.0167†
PD-L1^{CT}	1.010	1.005 to 1.015	<0.0001†
miRNA200c^{CT}	0.982	0.971 to 0.993	0.0020†
SALL4^{CT}	1.013	1.007 to 1.019	<0.0001†
**After backward selection**			
UICC (TNM) stage	1.712	1.065 to 2.751	0.0264†
SALL4^{CT}	2.163	1.033 to 4.530	0.0407†
PD-L1^{CT}	2.032	0.935 to 4.418	0.0734

NOTE: Abbreviations: HR, hazard ration; CI, confidence interval.*Log-rank *P* value corrected †Significant. All categorical covariates were transformed into numeric codes before they entered into the Cox model. Numeric codes are as follows: UICC-TNM stage: stage I = 1, stage II = 2, stage III = 3, stage IV = 4. Metastasis: N = 0, Y = 1. Tumor Volume, PD-L1, miRNA200c and SALL4 were divided into two groups by minimum *P* value cutoff, High group =1, Low group =0.

Supplementary table 4: The details and characteristics of each patients enrolled.

Cohort 1: HCC patients

Pts	Sex	Age	Operation Date	Survival	Follow-up	OS (months)	Metastasis	PrimarLiver lesion	Tumor Volume(cm^3)	HBV	HCV	HBsAg	HBsAb	HBeAg	HBeAb	HbcAb	Pathology Grading	T stage	N stage	M stage	Cirrhosis	Tumour thrombus	Cell type	Morphological subtype	Histopathology
NO.1	M		2007/1/17	Y	2013/9/1	80	N	Liver	9.375	Y	N	+	−	+	−	+	III	T3	N0	M0	N	N	Hepatocellular carcinoma	Nodular type	Trabecular
NO.2	M	67	2007/2/1	Y	2013/9/1	79	N	Liver	9.375	Y	N	+	−	−	+	+	I	T2	N0	M0	N	N	Hepatocellular carcinoma	Massive type	Trabecular, Pseudoglandular
NO.3	M	65	2007/2/12	N	2008/1/4	11	N	Liver	288	Y	N	+	−	−	+	+	III	T3	N0	M0	N	N	Hepatocellular carcinoma	Nodular type	Trabecular, Pseudoglandular
NO.4	M	58	2007/3/1	Y	2013/9/1	78	N	Liver	700	Y	N	+	−	−	+	+	III	T2	N0	M0	N	N	Hepatocellular carcinoma	Nodular type	Trabecular, Pseudoglandular
NO.5	M	49	2007/4/1	N	2008/3/24	11	N	Liver	43.1875	Y	N	+	−	−	+	+	II	T3	N0	M0	N	N	Hepatocellular carcinoma	Nodular type	Trabecular, Pseudoglandular
NO.6	M	71	2007/4/1	N	2008/6/30	14	N	Liver	352	Y	N	+	−	−	+	+	II	T3	N0	M0	N	N	Hepatocellular carcinoma	Massive type	Trabecular, Pseudoglandular
NO.7	M	70	2007/4/1	N	2008/5/31	13	Y	Liver	384	Y	N	+	−	−	+	+	II	T3	N1	M0	N	N	Hepatocellular carcinoma	Massive type	Trabecular, Pseudoglandular
NO.8	M	55	2007/4/1	N	2011/5/2	49	N	Liver	541.875	Y	N	+	−	−	+	+	I	T3	N0	M0	N	N	Hepatocellular carcinoma	Massive type	Trabecular, Pseudoglandular
NO.9	M	49	2007/6/1	N	2007/10/10	4	N	Liver	88	Y	N	+	−	−	+	+	III	T3	N0	M0	Y	N	Hepatocellular carcinoma	Nodular type	Trabecular, Pseudoglandular
NO.10	M	69	2007/7/1	N	2011/2/3	43	N	Liver	24.5	Y	N	+	−	−	+	+	III	T3	N0	M0	N	N	Hepatocellular carcinoma	Nodular type	Trabecular, Pseudoglandular
NO.11	F	44	2007/8/1	Y	2013/9/1	73	N	Liver	3.9375	Y	N	+	−	−	+	+	II	T2	N0	M0	Y	N	Hepatocellular carcinoma	Nodular type	Trabecular, Pseudoglandular
NO.12	M	51	2007/8/1	N	2010/1/19	29	N	Liver	83.1875	Y	N	+	−	−	+	+	III	T2	N0	M0	Y	N	Hepatocellular carcinoma	Nodular type	Trabecular, Pseudoglandular
NO.13	F	58	2007/8/1	Y	2013/9/1	73	N	Liver	7.8125	Y	N	+	−	−	+	+	II	T1	N0	M0	N	N	Hepatocellular carcinoma	Nodular type	Trabecular, Pseudoglandular
NO.14	M	50	2007/8/1	Y	2013/9/1	73	N	Liver	108	Y	N	+	−	−	+	+	II	T2	N0	M0	Y	N	Hepatocellular carcinoma	Nodular type	Trabecular, Pseudoglandular
NO.15	F	57	2007/8/1	N	2007/10/8	2	N	Liver	24.5	Y	N	+	−	−	+	+	III	T2	N0	M0	N	N	Hepatocellular carcinoma	Nodular type	Trabecular, Pseudoglandular
NO.16	M	52	2007/9/1	N	2013/6/9	69	N	Liver	7.8125	Y	N	+	−	−	+	+	II	T1	N0	M0	Y	N	Hepatocellular carcinoma	Nodular type	Trabecular, Pseudoglandular
NO.17	M	57	2008/1/1	N	2010/6/27	29	N	Liver	45.5	Y	N	+	−	−	+	+	III	T2	N0	M0	Y	N	Hepatocellular carcinoma	Massive type	Trabecular, Pseudoglandular
NO.18	M	43	2008/2/1	Y	2013/9/1	67	N	Liver	0.75	Y	N	+	−	−	+	+	II	T1	N0	M0	N	N	Hepatocellular carcinoma	Nodular type	Scirrhous
NO.19	F	48	2008/2/1	N	2010/3/23	25	N	Liver	198	Y	N	+	−	−	+	+	II	T3	N0	M0	N	N	Hepatocellular carcinoma	Nodular type	Trabecular, Pseudoglandular
NO.20	M	56	2008/3/1	N	2008/7/15	4	N	Liver	318.5	Y	N	+	−	−	+	+	III	T4	N0	M0	N	N	Hepatocellular carcinoma	Massive type	Trabecular, Pseudoglandular
NO.21	M	46	2008/3/1	N	2009/7/20	16	N	Liver	1521	Y	N	+	−	−	+	+	III	T3	N0	M0	N	N	Hepatocellular carcinoma	Massive type	Trabecular, Pseudoglandular
NO.22	M	52	2008/3/1	Y	2013/9/1	66	N	Liver	1568	Y	N	+	−	−	+	+	III	T3	N0	M0	N	N	Hepatocellular carcinoma	Massive type	Trabecular, Pseudoglandular
NO.23	M	53	2008/3/1	N	2008/7/17	4	N	Liver	5	Y	N	+	−	−	+	+	II	T1	N0	M0	N	N	Hepatocellular carcinoma	Massive type	Trabecular
NO.24	M	48	2008/3/1	N	2008/11/13	8	N	Liver	87.5	Y	N	+	−	−	+	+	II	T3	N0	M0	Y	N	Hepatocellular carcinoma	Nodular type	Trabecular
NO.25	M	56	2008/3/1	N	2008/9/3	6	N	Liver	786.5	Y	N	+	−	+	−	+	III	T3	N0	M0	N	N	Hepatocellular carcinoma	Nodular type	Trabecular, Pseudoglandular
NO.26	M	54	2008/3/1	N	2009/8/11	17	N	Liver	45.5625	Y	N	+	−	−	+	+	II	T2	N0	M0	N	N	Hepatocellular carcinoma	Massive type	Trabecular, Pseudoglandular
NO.27	M	57	2008/4/1	N	2009/3/12	11	N	Liver	3078	Y	N	+	−	−	+	+	III	T4	N0	M0	N	N	Hepatocellular carcinoma	Nodular type	Sarcomatous
NO.28	M	61	2008/7/1	Y	2013/9/1	62	N	Liver	1.6875	Y	N	+	−	−	+	+	II	T1	N0	M0	Y	N	Hepatocellular carcinoma	Diffuse type	Trabecular, Pseudoglandular
NO.29	F	66	2008/8/1	N	2011/1/18	29	N	Liver	40	Y	N	+	−	−	+	+	II	—	—	—	Y	N	Hepatocellular carcinoma	Massive type	Trabecular, Pseudoglandular
NO.30	M	49	2008/8/1	N	2009/1/14	5	Y	Liver	170.75	Y	N	+	−	−	+	+	III	T3	N0	M0	N	N	Hepatocellular carcinoma	Nodular type	Trabecular, Pseudoglandular
NO.31	M	68	2008/8/1	N	2008/9/4	1	N	Liver	486	Y	N	+	−	−	+	+	III	T3	N0	M0	Y	N	Hepatocellular carcinoma	Nodular type	Trabecular, Pseudoglandular
NO.32	M	48	2008/12/1	Y	2013/9/1	57	N	Liver	90.75	Y	N	+	−	−	+	+	I	T2	N0	M0	Y	N	Hepatocellular carcinoma	Massive type	Trabecular, Pseudoglandular
NO.33	M	48	2008/12/8	Y	2013/9/1	57	N	Liver	13.5	Y	N	+	−	−	+	+	II	T2	N0	M0	N	N	Hepatocellular carcinoma	Nodular type	Trabecular, Pseudoglandular
NO.34	F	65	2008/12/18	Y	2013/9/1	57	N	Liver	56	Y	N	+	−	−	+	+	II	T2	N0	M0	Y	N	Hepatocellular carcinoma	Nodular type	Trabecular, Pseudoglandular
NO.35	M	42	2008/12/22	N	2013/9/7	57	N	Liver	18	Y	N	+	−	−	+	+	II	T2	N0	M0	Y	N	Hepatocellular carcinoma	Nodular type	Trabecular, Pseudoglandular
NO.36	M	70	2008/12/22	Y	2013/9/1	57	N	Liver	72	Y	N	+	−	−	+	+	II	T2	N0	M0	Y	N	Hepatocellular carcinoma	Nodular type	Trabecular, Pseudoglandular
NO.37	M	39	2008/12/23	N	2010/5/9	17	N	Liver	320	Y	N	+	−	−	+	+	II	T3	N0	M0	Y	N	Hepatocellular carcinoma	Nodular type	Trabecular, Pseudoglandular
NO.38	M	70	2008/12/29	Y	2013/9/1	57	N	Liver	4	Y	N	+	−	−	+	+	II	T1	N0	M0	Y	N	Hepatocellular carcinoma	Massive type	Trabecular, Pseudoglandular
NO.39	M	51	2008/12/30	N	2010/5/7	17	N	Liver	245	Y	N	+	−	+	−	+	II	T3	N0	M0	N	N	Hepatocellular carcinoma	Nodular type	Trabecular, Pseudoglandular
NO.40	M	38	2008/12/30	N	2009/7/15	7	N	Liver	1470	Y	N	+	−	−	+	+	II	T3	N0	M0	Y	N	Hepatocellular carcinoma	Nodular type	Trabecular, Pseudoglandular
NO.41	M	37	2009/1/8	N	2009/7/19	6	Y	Liver	196	Y	N	+	−	−	+	+	II	T3	N0	M0	N	N	Hepatocellular carcinoma	Massive type	Trabecular, Pseudoglandular
NO.42	M	53	2009/1/16	N	2010/4/22	15	N	Liver	40	Y	N	+	−	−	+	+	III	T2	N0	M0	N	Y	Hepatocellular carcinoma	Nodular type	Trabecular, Pseudoglandular
NO.43	M	52	2009/1/21	N	2011/7/26	30	N	Liver	700	Y	N	+	−	−	+	+	II	T3	N0	M0	Y	N	Hepatocellular carcinoma	Nodular type	Trabecular, Pseudoglandular
NO.44	M	67	2009/1/23	N	2011/11/10	34	N	Liver	7.8125	Y	N	+	−	−	+	+	III	T1	N0	M0	N	N	Hepatocellular carcinoma	Nodular type	Trabecular, Pseudoglandular
NO.45	M	57	2009/2/9	N	2011-10-13	32	N	Liver	567	Y	N	+	−	−	+	+	III	T3	N0	M0	Y	N	Hepatocellular carcinoma	Nodular type	Trabecular, Pseudoglandular
NO.46	M	52	2009/2/10	Y	2013/9/1	55	Y	Liver	112.5	Y	N	+	−	−	+	+	III	T3	N0	M0	N	N	Hepatocellular carcinoma	Massive type	Trabecular, Pseudoglandular
NO.47	M	50	2009/2/27	Y	2013/9/1	55	N	Liver	665.5	Y	N	+	−	−	+	+	II	T3	N0	M0	N	Y	Hepatocellular carcinoma	Massive type	Trabecular, Pseudoglandular
NO.48	M	55	2009/2/27	Y	2013/9/1	55	N	Liver	126	Y	N	+	−	−	+	+	II	T2	N0	M0	Y	N	Hepatocellular carcinoma	Massive type	Trabecular, Pseudoglandular
NO.49	M	54	2009/3/9	Y	2013/9/1	54	N	Liver	9.375	Y	N	+	−	−	+	+	II	T2	N0	M0	Y	N	Hepatocellular carcinoma	Nodular type	Trabecular, Pseudoglandular
NO.50	M	51	2009/3/10	N	2009/8/26	5	N	Liver	15.75	Y	N	+	−	−	+	+	II	T2	N0	M0	N	N	Hepatocellular carcinoma	Nodular type	Trabecular, Pseudoglandular
NO.51	F	54	2009/3/11	N	2010/7/5	16	N	Liver	13.5	Y	N	+	−	−	+	+	II	T2	N0	M0	Y	N	Hepatocellular carcinoma	Nodular type	Trabecular, Pseudoglandular
NO.52	M	65	2009/3/16	N	2009/7/19	4	N	Liver	650	Y	N	+	−	−	+	+	II	T3	N0	M0	N	N	Hepatocellular carcinoma	Nodular type	Trabecular, Pseudoglandular
NO.53	M	67	2009/3/20	N	2009/9/30	6	Y	Liver	9.375	Y	N	+	−	−	+	+	II	T3	N0	M0	N	N	Hepatocellular carcinoma	Massive type	Trabecular, Pseudoglandular
NO.54	M	54	2009/3/25	Y	2013/9/1	54	N	Liver	270	Y	N	+	−	−	+	+	II	T3	N0	M0	N	N	Hepatocellular carcinoma	Nodular type	Trabecular, Pseudoglandular
NO.55	M	34	2009/3/27	Y	2013/9/1	54	N	Liver	24.5	Y	N	+	−	−	+	+	II	T2	N0	M0	N	Y	Hepatocellular carcinoma	Nodular type	Trabecular, Pseudoglandular
NO.56	M	50	2009/5/5	N	2011/9/14	28	N	Liver	364.5	Y	N	+	−	−	+	+	II	T3	N0	M0	N	N	Hepatocellular carcinoma	Massive type	Trabecular, Pseudoglandular
NO.57	M	63	2009/5/21	N	2012/2/21	33	N	Liver	249.64	Y	N	+	−	−	+	+	III	—	—	—	N	N	Hepatocellular carcinoma	Nodular type	Trabecular, Pseudoglandular
NO.58	M	45	2009/6/2	Y	2013/9/1	51	N	Liver	21.4375	Y	N	+	−	−	+	+	II	T2	N0	M0	N	N	Hepatocellular carcinoma	Massive type	Trabecular, Pseudoglandular
NO.59	M	49	2009/6/26	Y	2013/9/1	51	N	Liver	32	Y	N	+	−	−	+	+	III	T2	N0	M0	N	N	Hepatocellular carcinoma	Nodular type	Trabecular, Pseudoglandular
NO.60	M	55	2009/6/30	N	2011/9/16	27	Y	Liver	2176	Y	N	+	−	−	+	+	III	—	—	—	N	N	Hepatocellular carcinoma	Nodular type	Trabecular, Pseudoglandular
NO.61	M	53	2009/7/20	Y	2013/9/1	50	N	Liver	304	Y	N	+	−	−	+	+	III	T3	N0	M0	Y	N	Hepatocellular carcinoma	Nodular type	Trabecular, Pseudoglandular
NO.62	M	49	2009/7/22	Y	2013/9/1	50	N	Liver	936	Y	N	+	−	−	+	+	III	T3	N0	M0	N	Y	Hepatocellular carcinoma	Nodular type	Trabecular, Pseudoglandular
NO.63	F	40	2009/7/24	N	2010/5/3	10	N	Liver	1859	Y	N	+	−	−	+	+	II	T3	N0	M0	N	N	Hepatocellular carcinoma	Nodular type	Trabecular, Pseudoglandular
NO.64	M	59	2009/7/28	N	2010/11/12	16	N	Liver	281.25	Y	N	+	−	−	+	+	III	T3	N0	M0	Y	N	Hepatocellular carcinoma	Massive type	Trabecular, Pseudoglandular
NO.65	M	44	2009/7/28	N	2009/12/3	5	Y	Liver	1584	Y	N	+	−	−	+	+	II	T3	N0	M0	N	N	Hepatocellular carcinoma	Massive type	Trabecular, Pseudoglandular

NO.66	M	25	2009/7/31	Y	2013/9/1	50	N	Liver	143.6875	Y	N	+	−	−	+	+	II	T3	N0	M0	Y	N	Hepatocellular carcinoma	Massive type	Fibrolamellar
NO.67	M	40	2009/8/3	N	2011/9/24	25	N	Liver	100	Y	N	+	−	−	+	+	III	T2	N0	M0	Y	N	Hepatocellular carcinoma	Massive type	Trabecular, Pseudoglandular
NO.68	M	58	2009/8/26	Y	2013/9/1	49	N	Liver	93.75	Y	N	+	−	−	+	+	I	T2	N0	M0	Y	Y	Hepatocellular carcinoma	Massive type	Trabecular, Pseudoglandular
NO.69	M	63	2009/9/28	N	2009/11/21	2	Y	Liver	480	Y	N	+	−	+	−	+	III	T3	N0	M1	N	N	Hepatocellular carcinoma	Nodular type	Trabecular, Pseudoglandular
NO.70	M	26	2009/10/10	Y	2013/9/1	47	N	Liver	13.5	Y	N	+	−	−	+	+	II	T1	N0	M0	N	N	Hepatocellular carcinoma	Massive type	Trabecular, Pseudoglandular
NO.71	M	63	2009/10/29	N	2010/3/15	5	N	Liver	32	Y	N	+	−	−	+	+	II	T2	N0	M0	N	N	Hepatocellular carcinoma	Nodular type	Trabecular, Pseudoglandular
NO.72	M	58	2009/11/4	N	2012/5/24	30	N	Liver	27.5625	Y	N	+	−	−	+	+	I	T2	N0	M0	N	N	Hepatocellular carcinoma	Nodular type	Trabecular, Pseudoglandular
NO.73	M	73	2009/11/6	N	2011/6/6	19	N	Liver	68.75	Y	N	+	−	−	+	+	II	T4	Nx	Mx	Y	N	Hepatocellular carcinoma	Massive type	Trabecular, Pseudoglandular
NO.74	M	69	2009/11/6	N	2010/9/18	10	Y	Liver	48	Y	N	+	−	−	+	+	III	T3	N0	M0	N	N	Hepatocellular carcinoma	Nodular type	Trabecular, Pseudoglandular
NO.75	F	56	2009/11/11	Y	2013/9/1	46	N	Liver	20.25	Y	N	+	−	−	+	+	III	T3	N0	M0	N	N	Hepatocellular carcinoma	Nodular type	Trabecular, Pseudoglandular
NO.76	M	44	2009/11/11	N	2011/3/31	16	N	Liver		Y	N	+	−	−	+	+	II	T3	N0	M0	N	N	Hepatocellular carcinoma	Nodular type	Trabecular, Pseudoglandular
NO.77	M	62	2009/11/12	N	2012/10/10	35	N	Liver	87.5	Y	N	+	−	−	+	+	II	T3	N0	M0	N	N	Hepatocellular carcinoma	Nodular type	Trabecular, Pseudoglandular
NO.78	M	57	2009/11/17	N	2010/11/24	12	N	Liver	171.5	Y	N	+	−	−	+	+	II	T2	N0	M0	N	N	Hepatocellular carcinoma	Nodular type	Trabecular, Pseudoglandular
NO.79	M	59	2009/11/26	Y	2013/9/1	46	N	Liver	24.5	Y	N	+	−	−	+	+	II	T2	N0	M0	N	N	Hepatocellular carcinoma	Nodular type	Trabecular, Pseudoglandular
NO.80	M	70	2007/4/1	Y	2013/9/1	77	N	Liver	2.025	Y	N	+	−	−	+	+	III	T1	N0	M0	N	N	Hepatocellular carcinoma	Massive type	Trabecular, Pseudoglandular
NO.81	M	38	2007/7/1	Y	2013/9/1	74	N	Liver	2.25	Y	N	+	−	−	+	+	III	T1	N0	M0	N	N	Hepatocellular carcinoma	Nodular type	Trabecular, Pseudoglandular
NO.82	M	65	2008/4/1	N	2009/6/8	14	N	Liver	32	Y	N	+	−	−	+	+	I	T2	N0	M0	N	N	Hepatocellular carcinoma	Massive type	Trabecular, Pseudoglandular
NO.83	M	54	2008/11/1	N	2009/2/9	3	N	Liver	2.25	Y	N	+	−	−	+	+	II	T1	N0	M0	N	N	Hepatocellular carcinoma	Nodular type	Trabecular, Pseudoglandular
NO.84	M	47	2008/11/17	N	2012/6/6	43	N	Liver	4	Y	N	+	−	−	+	+	II	T1	N0	M0	N	N	Hepatocellular carcinoma	Nodular type	Trabecular, Pseudoglandular
NO.85	M	40	2008/12/1	Y	2013/9/1	57	N	Liver	15.75	Y	N	+	−	−	+	+	II	T2	N0	M0	N	N	Hepatocellular carcinoma	Nodular type	Trabecular, Pseudoglandular
NO.86	M	58	—	—	—	—	N	Liver	5	Y	N	+	−	−	+	+	I	T1	N0	M0	N	N	Hepatocellular carcinoma	Nodular type	Trabecular, Pseudoglandular
NO.87	F	56	—	—	—	—	N	Liver	5	Y	N	+	−	−	+	+	II	T1	N0	M0	Y	N	Hepatocellular carcinoma	Massive type	Trabecular, Pseudoglandular
NO.88	M	52	—	—	—	—	N	Liver	7.8125	Y	N	+	−	−	+	+	II	T1	N0	M0	Y	N	Hepatocellular carcinoma	Nodular type	Trabecular, Pseudoglandular
NO.89	M	56	—	—	—	—	N	Liver		Y	N	+	−	−	+	+	II	T2	N0	M0	Y	N	Hepatocellular carcinoma	Diffuse type	Trabecular, Pseudoglandular
NO.90	M	43	—	—	—	—	N	Liver	144	Y	N	+	−	−	+	+	II	T2	N0	M0	Y	N	Hepatocellular carcinoma	Nodular type	Trabecular, Pseudoglandular
NO.91	M	42	—	—	—	—	N	Liver	68.75	Y	N	+	−	−	+	+	II	T2	N0	M0	Y	N	Hepatocellular carcinoma	Massive type	Trabecular, Pseudoglandular
NO.92	M	61	—	—	—	—	N	Liver	13.5	Y	N	+	−	+	−	+	I	T2	N0	M0	Y	N	Hepatocellular carcinoma	Nodular type	Trabecular, Pseudoglandular
NO.93	M	54	—	—	—	—	N	Liver	179.5625	Y	N	+	−	−	+	+	III	T3	N0	M0	Y	N	Hepatocellular carcinoma	Nodular type	Trabecular, Pseudoglandular
NO.94	M	56	—	—	—	—	N	Liver	1166.4	Y	N	+	−	−	+	+	III	T3	N0	M0	Y	N	Hepatocellular carcinoma	Nodular type	Trabecular, Pseudoglandular
NO.95	M	57	—	—	—	—	N	Liver	126	Y	N	+	−	−	+	+	III	T3	N0	M0	N	Y	Hepatocellular carcinoma	Nodular type	Trabecular, Pseudoglandular
NO.96	M	63	—	—	—	—	N	Liver	530.55	Y	N	+	−	−	+	+	III	T3	N0	M0	N	N	Hepatocellular carcinoma	Nodular type	Trabecular, Pseudoglandular
NO.97	M	58	—	—	—	—	N	Liver	660	Y	N	+	−	−	+	+	III	T3	N0	M0	N	N	Hepatocellular carcinoma	Nodular type	Trabecular, Pseudoglandular
NO.98	M	53	—	—	—	—	N	Liver	554	Y	N	+	−	−	+	+	III	T3	N1	M0	N	N	Hepatocellular carcinoma	Nodular type	Trabecular, Pseudoglandular

Cohort 2: LC patients

Pts	Diagnosis	Sex	Age	Metastasis	Pathology Grading	T	N	M	AJCC-TNM
NO.1	Cirrhosis	M	47	——	——	——	——	——	——
NO.2	Cirrhosis	M	38	——	——	——	——	——	——
NO.3	Cirrhosis	M	49	——	——	——	——	——	——
NO.4	Cirrhosis	M	43	——	——	——	——	——	——
NO.5	Cirrhosis	M	43	——	——	——	——	——	——
NO.6	Cirrhosis	M	49	——	——	——	——	——	——
NO.7	Cirrhosis	M	53	——	——	——	——	——	——
NO.8	Cirrhosis with hyperplasia	M	60	——	——	——	——	——	——
NO.9	Cirrhosis with hyperplasia	M	57	——	——	——	——	——	——
NO.10	Cirrhosis with hyperplasia	F	48	——	——	——	——	——	——
NO.11	Cirrhosis with hyperplasia	F	63	——	——	——	——	——	——
NO.12	Cirrhosis with hyperplasia	F	24	——	——	——	——	——	——
NO.13	Cirrhosis with hyperplasia	F	56	——	——	——	——	——	——

Cohort 3: Healthy control

Donor	Diagnosis	Sex	Age	Metastasis	Pathology Grading	T	N	M	AJCC-TNM
NO.1	Normal liver	M	adult	——	——	——	——	——	——
NO.2	Normal liver	M	adult	——	——	——	——	——	——
